# A broadband photodetector based on Rhodamine B-sensitized ZnO nanowires film

**DOI:** 10.1038/s41598-017-11154-8

**Published:** 2017-09-12

**Authors:** Zheng Qi Bai, Ze Wen Liu

**Affiliations:** Institute of Microelectronics, Tsinghua University, Beijing, 100084 China

## Abstract

A broadband photodetector has been developed on the basis of ZnO nanowires (NWs)/Rhodamine B (RhB) hybrid system. The device is fabricated by spraying NWs on to gold interdigital electrodes followed by modifying the NWs via an RhB solution-casting process. Measurements show that the as-fabricated device demonstrates photoresponsivity ranging from 300 nm to 700 nm with a bandwidth as large as 400 nm. The role of the dye sensitizer adsorbed on the surface of NWs is modeled to alter the transportation path of photo-generated carriers. The calculations based on the measurements reveal that the device exhibits a prominent responsivity in the interested band with maximum responsivity of 5.5 A/W for ultraviolet (UV) light and 3 A/W for visible (VIS) light under 8 V bias, respectively. The sensitization not only widens the response spectrum with external quantum efficiency leaping up to 771% at VIS but also improves UV responsivity with maximum 51% enhancement. From the time–dependent photo-current measurement, it is found that the response time (rise and decay times in total) of the device largely reduced from 17.5 s to 3.3 s after sensitization. A comparison of the obtained photodetector with other ZnO-based photodetectors is summarized from the view point of responsivity and bandwidth.

## Introduction

Continuous research interest has been attracted to zinc oxide (ZnO) nanostructures over twenty years for its widespread potential applications in ultraviolet (UV) photodetectors^1, 2^, field effect transistors (FET)^3, 4^, laser diodes (LDs)^5, 6^, and light emitting diode (LED)^7^ owing to its unique properties such as wide-bandgap (~3.37 eV), larger specific surface area, carrier confinement in two or three dimensions, high exciton binding energy (approximately 60 meV), and chemical stability^8–12^. Among various nanostructures, wurtzite (hexagonal) structured ZnO nanowires (NWs) have been regarded as one kind of extremely promising building blocks for fabricating high-gain optoelectronic nano-devices^13–16^ due to its excellent properties, relatively simplistic preparation process, and the possibility of surface modification.

To date, the broadband photodetectors covering the visible band (VIS, wavelength ranges from 390 nm to 780 nm) have gradually become an important research domain due to the great demands of environmental monitoring^17^, composition analysis^18^, photometric measurement^19^, communication^20^, and integrated optical system such as micro-spectrometer^21^. To broaden the response bandwidth and overcome the “visible-blind” characteristics of low absorbance and weak responsivity of ZnO NWs for VIS light has become an interesting research focus recently^22, 23^.

A variety of studies on strategies for widening the photosensitive bandwidth of ZnO have been reported, including ZnO NWs doping with trivalent elements like Al, Ga and In^24, 25^ and co-growth with narrow bandgap (2.0 eV~2.4 eV) materials CdS or Cu_2_O nanostructure^26, 27^. All these methods need complicated facilities or demand carefully chemical synthesis process control, and still to be developed for practical application. Dye sensitization provides an alternative method to widen the material response spectrum. It was firstly proposed by O’regan B^28^ in 1991, and then reportedly used in solar cells to enhance its efficiency^29–31^. The attractions to this method are its cost effectiveness and easy process handling, regardless of longtime material stability issues. The possible breakthrough of this method is using novel low toxicity organic dye material and function material combination for special potential application such as photodetectors.

In this work, we report a novel broadband photodetector using ZnO NWs surface-modified by an organic dye called Rhodamine B (RhB) as function materials. We begin by introducing the fabrication process and measurement methods. The active area of the broadband photodetector consists of pre-fabricated interdigital electrodes covered by dispersive ZnO NWs, which are sensitized by dye molecule. And then, we present and discuss our experimental results. Optic-electronic measurements show that photodetectors made of RhB-sensitized ZnO NWs have a wide response spectrum from 300 nm to 700 nm with a high responsivity and bandwidth as large as 400 nm. A model was established to explain the mechanism of broadening response spectrum of ZnO/RhB hybrid system, in which carriers transport path and photoelectric property are greatly affected by the surface modification of dye and the surface defect states of ZnO NWs. The optoelectronic and time-dependent photo response properties of the photodetector were measured at certain UV and VIS wavelength, respectively. Based on the measurement and the model, further calculations of responsivity and the external quantum efficiency (EQE) were performed. An increase of approximate 2 to 26 times in the responsivity for VIS light is obtained after sensitization, which corresponds to a 771% increment of the EQE at the maximum point in VIS band. Meanwhile, the EQE in UV band is also enhanced with a maximum value of 51%. From the time–dependent photo-current measurement, it shows that the response time (rise and decay times in total) of the device is significantly reduced from 17.5 s to 3.3 s for UV and and 16 s to 4.3 s for VIS light by comparison with the devices made of only pure ZnO NWs, respectively. Finally, we conclude that the demonstration of the as-fabricated device with a broad bandwidth and enhanced responsivity envisions a feasible and cost-effective method to employ organic sensitizer for the broadband photodetector.

## Results and Discussion

**Figure 1:**
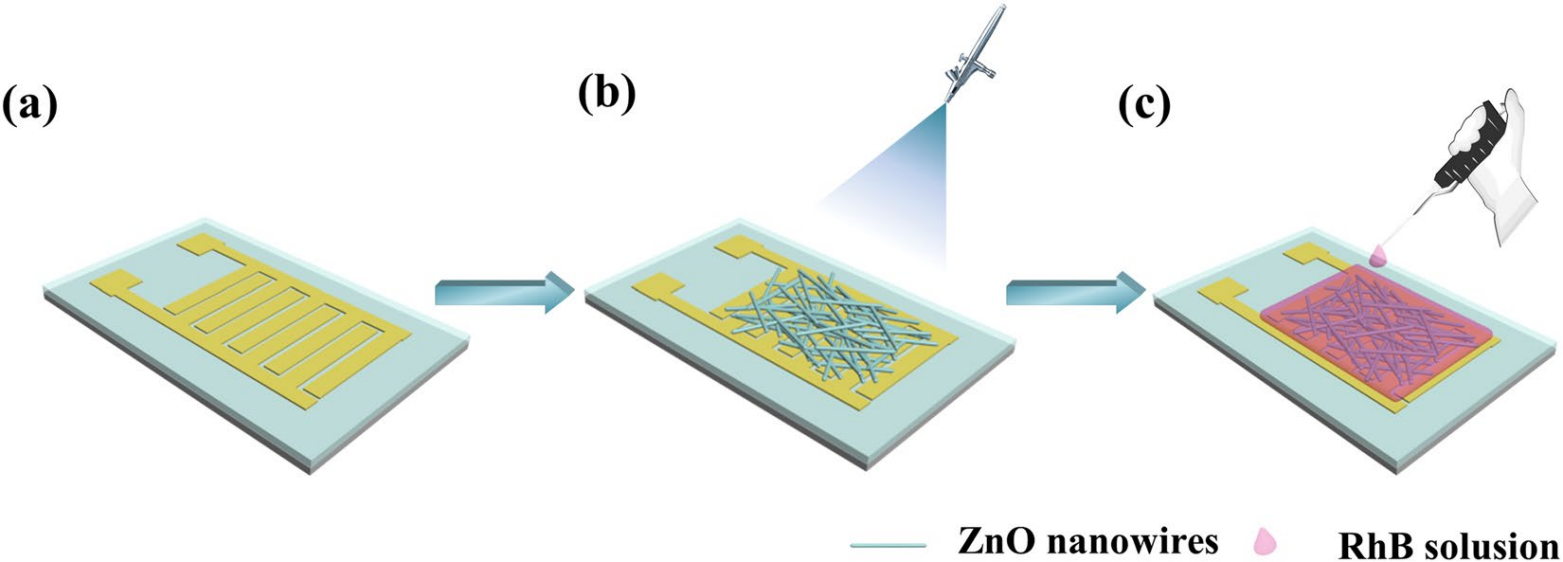
Fabrication process of the proposed broadband photodetector.

The fabrication process of the device is depicted schematically in Fig. 1. The device can be fabricated by spraying NWs on to gold interdigital electrodes followed by modifying NWs via an RhB solution-casting process. The specific details are described in the method section.

**Figure 2:**
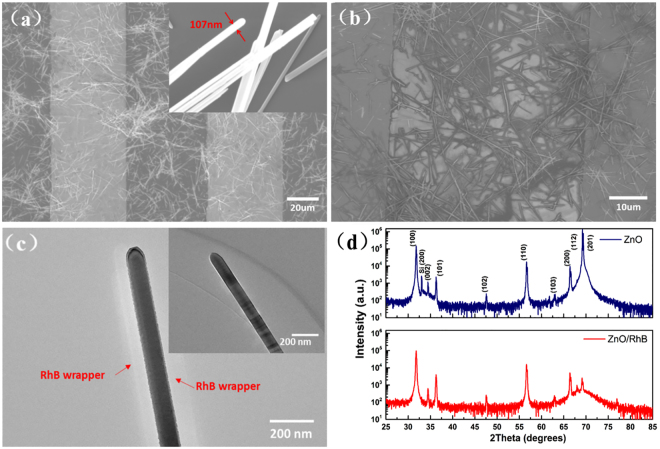
(**a**) SEM image of ZnO NWs films prepared on the interdigital electrodes, the inset shows the pure single ZnO NWs. (**b**) SEM image of RhB sensitized ZnO NWs film prepared on the interdigital electrodes. (**c**) TEM image of single ZnO NW coated by RhB, the inset shows the pure single ZnO NWs. (**d**) XRD pattern of ZnO NWs and RhB sensitized ZnO NWs films.

The morphology analysis and material characteristics of ZnO NWs are shown in Fig. 2. Figure 2(a) shows the SEM image of ZnO NWs before sensitization. The measured mean diameter of ZnO NWs is about 100 nm and the morphology of porous is beneficial to dye penetration. The ZnO NWs can interpenetrate each other densely and form stacked network structures to bridge the gap between adjacent interdigital electrodes. The SEM image of ZnO NWs after sensitization is shown in Fig. 2(b). Dye combined with NWs well without agglomeration and all of the morphology, uniformity, and distribution have not been changed. As shown in the picture, the photosensitive film is composed of NWs infiltrated by the crystallization of sensitized-dye. Figure 2(c) shows an enlarged TEM image, which reveals the structure of single RhB-coated ZnO NWs clearly. Compared with the pure ZnO NWs with a diameter of 107 nm displayed in the inset, the faint bright cladding circled the single-NWs may be crystalline RhB. It is obvious that both ZnO NWs and ZnO NWs/RhB hybrid photosensitive film are uniform, compact and densely packed. The XRD pattern of ZnO NWs and the ZnO NWs/RhB hybrid system were characterized by XRD (Cu K_α_, λ = 0.15406 nm), which is shown in Fig. 2(d). All the sharp diffraction peaks can be well indexed to hexagonal ZnO NWs with wurtzite structure. By comparing the intensity of these diffraction peaks, the introduction of RhB slightly decreased the crystallinity of ZnO NWs and two relatively weak diffraction signals of RhB appeared.

**Figure 3:**
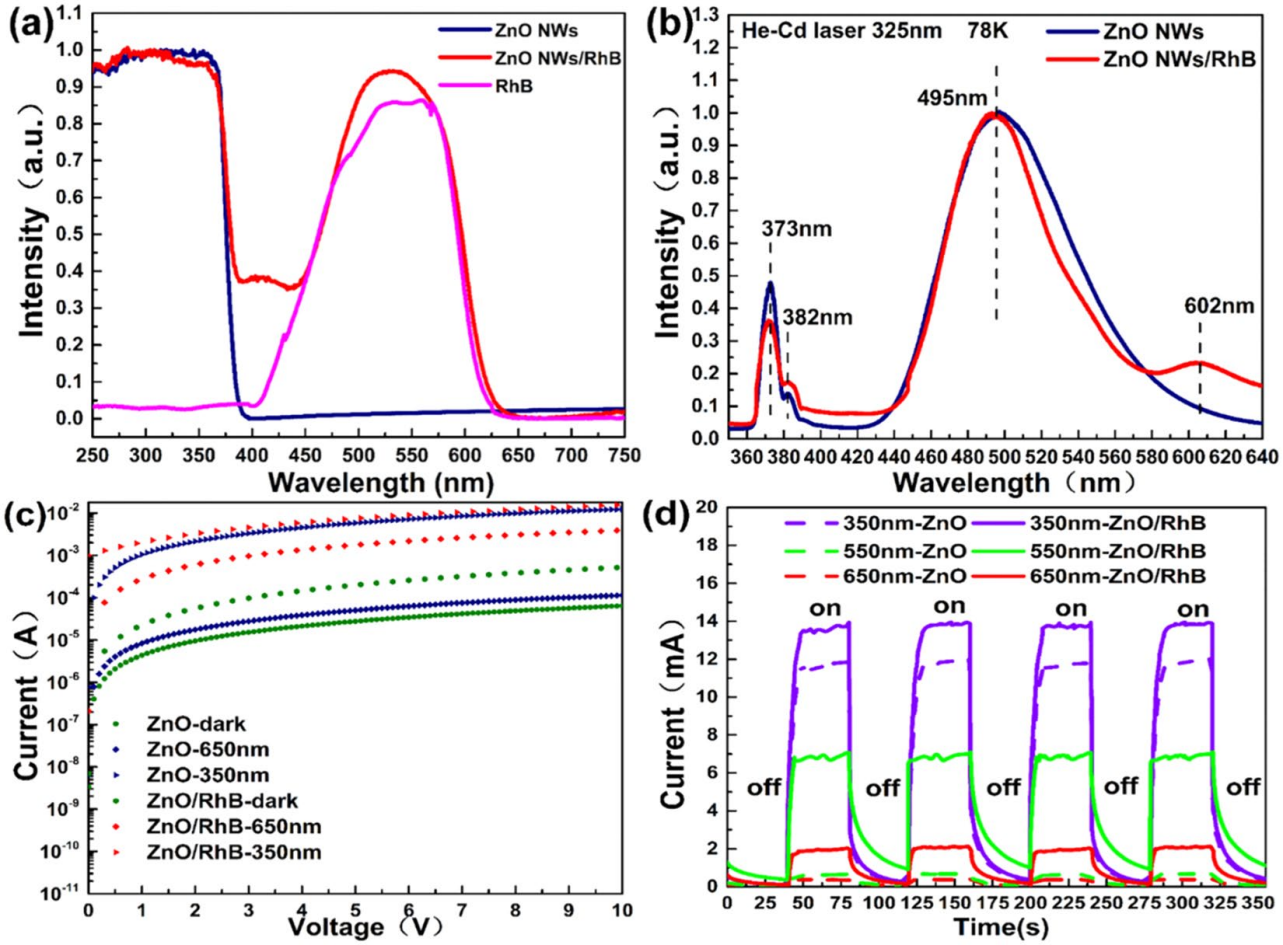
(**a**) Absorption spectra of ZnO NWs, ZnO NWs/RhB, and pure RhB films. (**b**) Low-temperature photoluminescence spectra of ZnO NWs and ZnO NWs/RhB films. (**c**) Measured current-voltage (I-V) characteristics of the photodetectors with or without sensitized-dye. (**d**) Time-dependent measurements of photo-response with UV (2.4 mW@350 nm) light and different visible (2.8 mW@550 nm, 2.95 mW@650 nm) light over four cycles.

The absorption spectra (from 200 to 700 nm) and photoluminescence (PL) spectra are plotted in Fig. 3(a). The result indicates that RhB in the hybrid system has a significant role in broadening the absorption spectrum. An obvious cut off phenomenon of ZnO NWs film at nearly 400 nm appears while the effective absorption peak of RhB concentrates ranging from 450 nm to 600 nm approximately.

The low-temperature (78 K) photoluminescence of the as-prepared ZnO NWs and ZnO NWs/RhB hybrid films were measured with an excitation power of 0.32 W/cm^2^, as shown in Fig. 3(b). Non-radiative recombination can be suppressed significantly under low temperature, while a slight red shift of the emission peaks may be inevitable cause of the contraction band phenomenon. There are three emission peaks: the ultraviolet emission at 373 nm should correspond to the intrinsic transition between the band gap of ZnO, a weak shoulder peak at 382 nm is the first-order phonon line. The blue-green strong wide VIS emission with the center wavelength of approximately 495 nm and full width at half maximum of 95 nm is possibly associated with deep-level defects and the singly ionized oxygen vacancy in ZnO, resulting from the recombination of photo-generated hole with the single ionized charge state of this defect^32^. As the result of sample infiltrated by RhB shows, a new wide emission with the peak wavelength of 602 nm appears.

The quantitative measurements of the response current under different specific wavelengths were conducted by the semiconductor characterization system (Keithley 1500-SCS) under dark condition, illuminated by UV light (peak wavelength 350 nm, with power 2.4 mW) and VIS light (peak wavelength 650 nm with power 2.95 mW). The current-voltage (I-V) characteristics of the photodetectors with or without dye sensitization are shown in Fig. 3(c). The measurement results prove that the obvious role of dye sensitization in increasing responsivity to VIS light band is effective. The time-dependent measurements of photo-response were employed to research the rise and decay time with excitation (power: 2.4 mW @350 nm, 2.8 mW@550 nm, 2.95 mW@650 nm) switched on and off for the same time intervals at 8 V bias. As shown in Fig. 3(d), the photocurrent could be reversibly modulated by irradiation, increase rapidly and remain stable under the steady illumination at different wavelengths. Drift in the dark current regarding time was observed, which can be explained by persistent photoconductivity effects.

**Figure 4:**
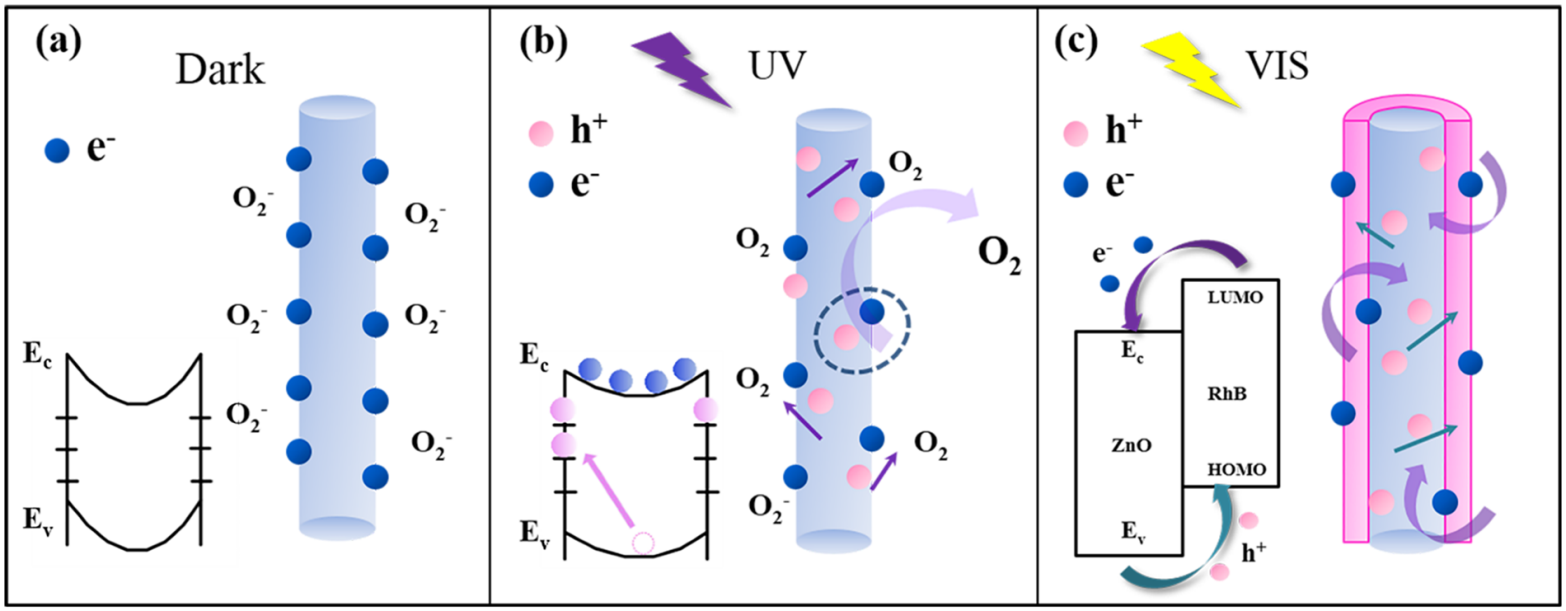
(**a**) Illustrative schematic of ZnO NWs in dark condition. The left drawing in (**a**) illustrates the schematic of energy band diagrams of ZnO NWs, showing band-bending and trap states. The right drawing shows oxygen molecules adsorbed at the surface of NWs that capture free electrons forming a low conductivity depletion layer near the surface. (**b**) Illustrative schematic of ZnO NWs in UV illumination condition. Photo-generated holes migrate to the surface and are trapped, while unpaired photo-generated electrons are pumped to a conducting band of ZnO contributing to the photocurrent. (**c**) Dye-sensitized mechanism. The left drawing in (**c**) illustrates an energy level under VIS illumination of ZnO/RhB hybrid system.

The mechanism to explain the photosensitive characteristic of ZnO NWs and the amazing effect of dye molecules in broadening light responsivity spectrum are shown in Fig. 4, revealing the illustrative schematic of pure ZnO NWs and dye-sensitized ZnO NWs. As shown in Fig. 4(a), Oxygen molecules are naturally adsorbed onto the surface of ZnO NWs due to the existence of surface dangling bonds and free electrons from NWs are then captured in dark conditions, leading to band bending, which can be expressed in equation (1). Therefore, a depletion layer with low conductance and a high barrier comes into form near the surface of ZnO NWs, resulting in a low dark current. Figure 4(b) describes the condition in illumination with UV light. Driven by the internal potential, photogenerated holes are migrated to the surface of NWs and captured by the surface hole-traps, leading to a reduction of the depletion barrier thickness and the height of the barrier. The reaction process of desorption from oxygen adsorbents is shown in equation (2). In the meantime, the unpaired photo-generated electrons are pumped to conducting band from valence band, which increases the conductivity of ZnO NWs and generates a significant photocurrent.

1 $${{\rm{O}}}_{{\rm{2}}}({\rm{g}})+{{\rm{e}}}^{-}\to {{\rm{O}}}^{2-}({\rm{ad}})$$

2 $${{\rm{h}}}^{+}+{{\rm{O}}}^{2-}({\rm{ad}})\to {{\rm{O}}}_{{\rm{2}}}({\rm{g}})$$

This hole-trapping mechanism through the adsorption and desorption of oxygen molecule in ZnO NWs accounts for the enhancement of the photo-response for UV light, but the responsivity decreases with the increment of wavelength especially above 500 nm. In our research, dye RhB was adopted as the sensitizer in order to alter the transfer pathway of the photo-generated carriers. The dye-sensitized mechanism is displayed in Fig. 4(c), electrons are activated from the highest occupied molecular orbital (HOMO ~ −5.3 eV) to the lowest unoccupied molecular orbital (LUMO ~ −3.2 eV) after the assimilation of low-frequency photons corresponding to a lower energy band of VIS light, which subsequently injected into the bottom of the conducting band (E_c_ ~ −4.2 eV) of ZnO. At the same time, holes migrate from the top of the valence band (E_v_ ~ −7.6 eV) of ZnO to HOMO of RhB. Unavoidably, the carriers transfer between the HOMO of dye and the E_c_ of ZnO may slightly increase the dark current after sensitization.

**Figure 5:**
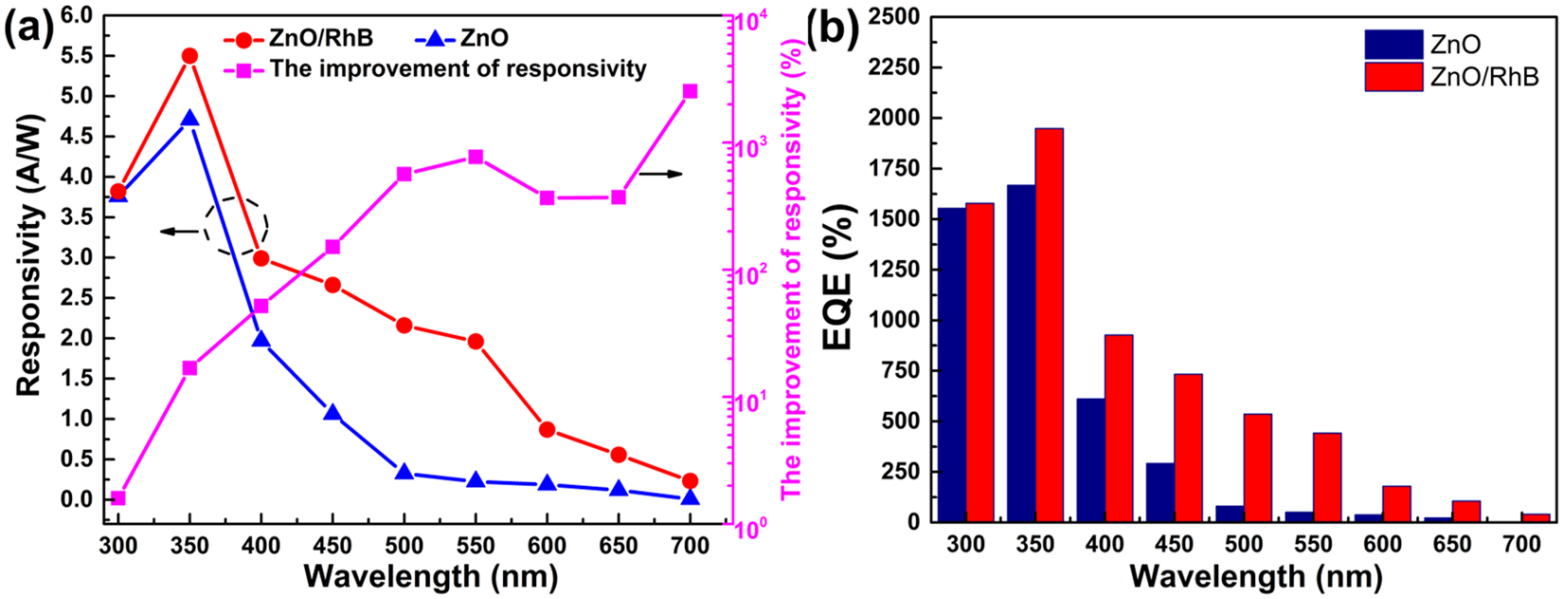
(**a**) Responsivity and its improvement of the photodetector versus wavelength. (**b**) External quantum efficiency (EQE) of the photodetector versus wavelength.

The responsivity as well as EQE among a wide bandwidth of the photodetector were further studied and analyzed. The responsivity and its improvement of the broadband photodetector as a function of wavelength at 8 V bias are shown in Fig. 5(a). The responsivity is used to describe the photoelectric conversion capability of the photodetector, which is expressed as R = I/P, where R is responsivity, I is photocurrent and P is incident light power. The measurement results indicate that the responsivity rises firstly and then decreases with the increase of wavelength. Compared with the device with only ZnO NWs, an extreme increase on responsivity (maximum 3 A/W at 550 nm) at VIS band has been achieved for our broadband photodetector modified and sensitized by the dye. Specifically, the responsivity of the device with ZnO NWs decreases sharply as the increase of wavelength and shows incredibly low response characteristic above 450 nm. As displayed, an improvement of approximate 2 to 26 times in responsivity to VIS and a maximum 51% growth to UV were obtained.

EQE is equal to responsivity multiplied by photon energy described as EQE = R × hc/eλ, where R is responsivity calculated above, h is Planck constant, c is the velocity of light, e is the charge of an electron and λ is the wavelength of incident light. Figure 5(b) manifests that an obvious improvement on EQE (maximum up to 771% for VIS light) can be observed for this broadband photodetector based on dye-sensitized ZnO NWs film, illustrating the excellent response characteristics of our novel device both in UV and VIS bands.

Besides responsivity and EQE, the specific detectivity is another one crucial figure-of-merits for the photodetector, which is usually used to describe the minimal detectable signal^33^.

3 $$\,{\boldsymbol{D}}* =\sqrt{{\boldsymbol{s}}\times {\rm{\Delta }}{\boldsymbol{f}}}\frac{1}{{\boldsymbol{NEP}}}$$

4 $${\bf{N}}{\bf{E}}{\bf{P}}=\frac{\sqrt{{{\boldsymbol{i}}}_{{\boldsymbol{n}}}^{{\bf{2}}}}}{{{\boldsymbol{R}}}_{{\boldsymbol{\lambda }}}}$$

where ***s*** is the effective area of the detector with the unit of cm^**2**^, Δ*f* is bandwidth, **NEP** is the noise equivalent power, $\sqrt{{{\boldsymbol{i}}}_{{\boldsymbol{n}}}^{{\bf{2}}}}$ is the measured dark current and ***R***_*λ*_ is the responsivity. In particular, at the operating bias of 8 V, the D* of the photodetector was 2.34 × 10^11^ cmHz^**1/2**^W^**−1**^. The results imply that our photodetector has a potential for wide spectrum detection with simpler process and high performance.

**Figure 6:**
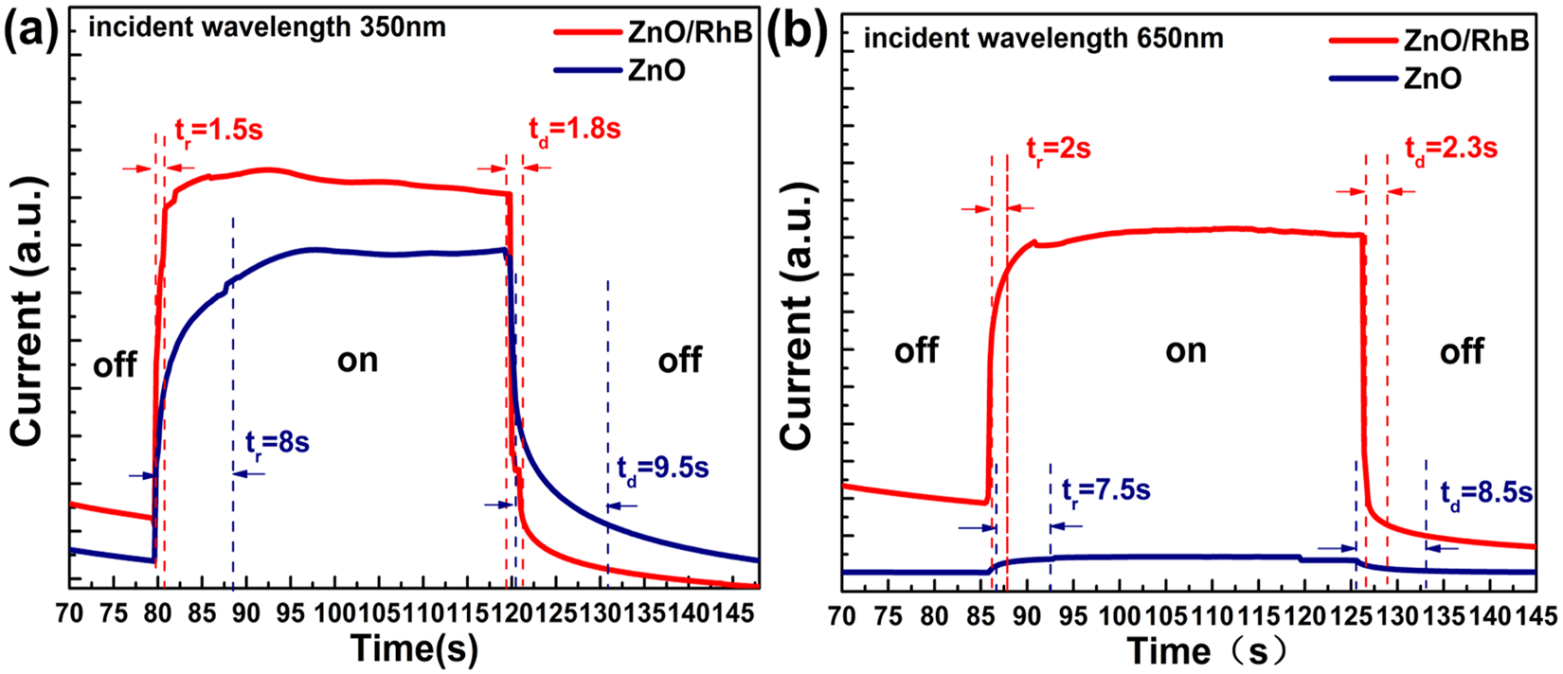
(**a**) Response current of the photodetector with switch on and off under 350 nm incidence, the bias voltage was 8 V and irradiance was 16 mW/cm^2^. (**b**) Response current of the photodetector with switch on and off under 650 nm incidence, bias voltage was 8 V and irradiance was 19.6 mW/cm^2^.

Furthermore, the use of RhB decreases the response time by reducing the recombination of photo-generated carriers prominently. According to Figs 3(d), 6(a) and (b) show the details on rising and decay time curve of the broadband photodetector at two kinds of representative wavelength with similar light power density at 8 V bias. The rise time (t_r_) is defined as the range that photocurrent rises from 10% to 90% of its maximum. The decay time (t_d_) is defined similarly. As shown in Fig. 6, the response time (the sum of the rise time and decay time) of the device with dye sensitization significantly reduced from 17.5 s to 3.3 s for UV light and 16 s to 4.3 s for VIS light, respectively, by comparison with devices with pure ZnO NWs only. More in details, the UV photocurrent rise quickly and reach saturation with the t_r_ of 1.5 s, while the photocurrent gradually decreased and recovered to the initial state with the t_d_ of 1.8 s. Rise and decay time of the same order were demonstrated when using VIS incident wavelength, showing a t_r_ of 2 s and t_d_ of 2.3 s respectively. But without ignorance, an exciting reset time (less than 800 ms) of ZnO NWs UV photodetector has been achieved by utilizing Schottky contact and surface functionalization with polymers^34^.

**Figure 7:**
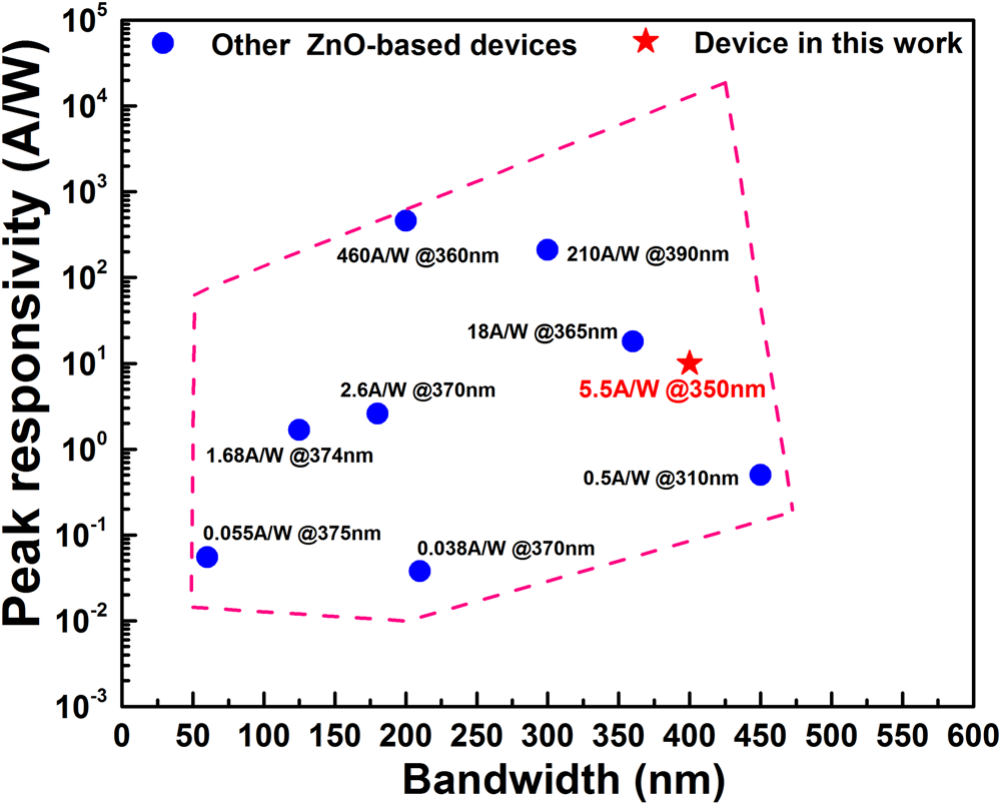
Performance comparison of different ZnO based photodetectors^35–43^.

Figure 7 exhibits the performance comparison of different ZnO based photodetectors by plotting the peak responsivity as a function of detection bandwidth. A result of a 400 nm response bandwidth at least and 5.5 A/W peak responsivity was realized by our broadband photodetector based on dye-sensitized ZnO NWs film with a simple fabrication process. As displayed in above diagram, although a ZnO-based photodetector with high (about 460 A/W) responsivity has been achieved before, its response bandwidth is about 200 nm. Other ZnO-based photodetectors have their own advantages in terms of bandwidth and responsivity severally. More works need continued to further promote the responsivity of our device in the future.

## Conclusion

In conclusion, a novel broadband photodetector based on dye-sensitized ZnO NWs film has been proposed, fabricated and discussed. The RhB sensitization mechanism has been investigated. Through the sensitization mechanism, it can be beneficial to increase the responsivity and EQE for VIS light, improves UV responsivity, and also optimize the time resolved characteristics. The research results show that the broadband photodetectors sensitized by the organic dye RhB molecules exhibit a prominent photo-response for a broadband light (300 to700 nm) with the approximate responsivity of 5.5 A/W to UV light and maximum 3 A/W to VIS light at 8 V bias, respectively. By comparison with other ZnO-based photodetectors which have been reported, the response bandwidth of our device reaches up to 400 nm. According to the results of this work, the demonstrated method may be a promising, simple, cost-effective, and feasible technological means to realize broadband photo detection. More theoretical and experimental studies are necessary to improve the performances of our device further.

## Methods

### Device fabrication

The ZnO/RhB hybrid broadband photodetector was fabricated via the following processes in an ultra-clean environment. An N-type (100) oriented silicon wafer was used as the substrate, and a 300 nm thick silicon dioxide (SiO_2_) dielectric layer was thermally grown on the substrate for insulation protection. The Au interdigital electrodes with same finger width and gap width of 50 μm were fabricated by standard photolithography, Titanium/Gold (Ti/Au, 20 nm/100 nm) RF sputtering, and the following lift-off process. Then the ZnO NWs suspension was sprayed on interdigital electrodes to form a uniform photosensitive layer. The dispersion solution was prepared by dispersing ZnO with an approximate average diameter of 100 nm in ethanol with the ratio of 1 g (NW): 400 ml (E) achieving a concentration of 2.5 g/L and then ultra-sonicated for 2 h before using. Then the interdigital electrodes coated with ZnO NWs was heated at 60 °C for 30 s and annealed in N_2_ atmosphere at 350 °C for 8 min to achieve a better ohmic contact between metal electrodes and ZnO NWs. Finally, the RhB solution with a concentration of 0.1 mg/ml was dropped onto the ZnO NWs under the background of heating at 80 °C for 30 s. The illuminated active area of the device is 5 × 3 mm^2^, and the thickness of ZnO NWs film is about 1 μm.

### Measurement details

The morphology of photosensitive materials is characterized by scanning electron microscope (SEM) and transmission electron microscope (TEM). The crystalline structure of ZnO NWs and ZnO NWs/RhB hybrid film were examined by X-ray diffraction (XRD) with scanning degree ranging from 25°to 85°. The absorption spectra of ZnO NWs and ZnO NWs/RhB hybrid films were recorded by the photometer. The low-temperature photoluminescence (PL) measurement was performed at 78 K using 325-nm He-Cd laser as the excitation source.

Current–voltage (I-V) characteristic curve was recorded by sweeping the bias voltage from 0 V to 10 V across the electrodes using the Keithley 1500SCS semiconductor characterization system. All devices were kept in the darkroom for more than 24 h to stabilize before measuring dark current. The photocurrent was tested by illuminating the active area with discrete wavelengths through a monochromator (Triax190) from a Xenon Light Source at 8 V bias. All measurements were performed in the atmosphere at room temperature except for the low-temperature PL.
